# Biocompatibility of 3D-Printed Dental Resins: A Systematic Review

**DOI:** 10.7759/cureus.51721

**Published:** 2024-01-05

**Authors:** Jayant Prakash, Mahesh Shenoy, Abdulmajeed Alhasmi, Azzam A Al Saleh, Shivakumar G C, Sahana Shivakumar

**Affiliations:** 1 Department of Prosthodontics, Dental Institute, Rajendra Institute of Medical Sciences, Ranchi, IND; 2 Department of Oral and Maxillofacial Surgery and Diagnostic Sciences, College of Medicine and Dentistry, Riyadh Elm University, Riyadh, SAU; 3 Department of Periodontics, King Salman Hospital, Ministry of Health, Riyadh, SAU; 4 Department of Dentistry, Ministry of Health, Riyadh, SAU; 5 Department of Oral Medicine and Radiology, People’s College of Dental Sciences and Research Centre, Bhopal, IND; 6 Department of Public Health Dentistry, People’s College of Dental Sciences and Research Centre, Bhopal, IND

**Keywords:** cell viability, mechanical properties, cytotoxicity, additive manufacturing, systematic review, dental resins, 3d printing, biocompatibility

## Abstract

Background: The biocompatibility of 3D-printed dental resins has become a critical concern in modern dentistry due to the increasing utilization of additive manufacturing (AM) techniques in dental applications. These resins serve as essential materials for fabricating dental prostheses, orthodontic devices, and various dental components. As the clinical adoption of 3D printing in dentistry grows, it is imperative to comprehensively assess the biocompatibility of these materials to ensure patient safety and dental treatment efficacy. This systematic review aimed to evaluate the existing body of literature on the biocompatibility of 3D-printed dental resins, thereby providing valuable insights into the potential biological risks associated with their use.

Methods: The search strategy to identify relevant papers was implemented across PubMed/MEDLINE, Scopus, Web of Science, Embase, Cochrane Library, CINAHL, and Google Scholar to identify relevant studies. Study selection was not limited to any particular timeframe of publishing. The revised CONSORT criteria were used to ascertain the authenticity and dependability of the review's outcomes. Comprehensive screening and eligibility assessment processes were conducted to select studies meeting predefined criteria. Biocompatibility-related parameters, including toxicity, mechanical properties, cell viability, and other relevant outcomes, were analyzed across selected studies using a standardized variable extraction protocol.

Results: A total of 9 studies were included in the systematic review. The findings encompassed various aspects of biocompatibility assessment, including material composition, mechanical properties, cell viability, and cytotoxicity. Some studies revealed significant improvements in flexural strength and cell viability with specific resin formulations, demonstrating their potential for enhanced clinical utility. Conversely, certain resins exhibited cytotoxicity, while others displayed promising biocompatibility profiles.

Conclusion: As per the assessed findings, material composition, post-processing techniques, and manufacturing methods emerged as critical factors influencing biocompatibility outcomes. While some resins exhibited favorable biocompatibility profiles, others raised concerns due to cytotoxicity. These findings emphasize the need for careful consideration when selecting and implementing 3D-printed dental resins, with a focus on materials engineering and comprehensive biocompatibility testing. Further research is warranted to elucidate the long-term biocompatibility and clinical implications of these materials.

## Introduction and background

The swift advancement of 3D printing technology has transformed several industries, including dentistry, as it makes it possible to create complex, patient-specific dental components [1]. In particular, 3D-printed dental resins have gained substantial attention due to their potential for creating customized dental prostheses, orthodontic devices, and restorative materials [2]. However, alongside the remarkable innovations in this domain, there arises a paramount concern that demands careful scrutiny: the biocompatibility of these 3D-printed dental resins [3-4].

A plethora of 3D printing methodologies have emerged in the realm of dentistry, each tailored to specific dental applications [5,6]. The judicious selection of 3D printing materials hinges on the intended function of the final dental construct. Dental restorations, for instance, necessitate materials endowed with robust mechanical attributes and protracted biodegradability, prerequisites to withstand the formidable masticatory forces. Moreover, seamless integration with oral tissues is indispensable for the success of dental restorations [7]. When it comes to dental prosthesis design, special attention to important requirements like better mechanical and physical properties, compatible biocompatibility, ease of handling, and cost-effectiveness is still crucial, especially when it comes to temporary crowns. The most popular substrates for the additive fabrication of dental prostheses are polymer-based materials, primarily resins. On the other hand, composite resin-based materials are characterized by the combination of filler particles, coupling agents, resin matrices, and catalysts that are skillfully combined to produce homogenous blends [8].

In the perpetual pursuit of optimizing provisional dental resins, with a paramount emphasis on augmenting durability and biocompatibility, a medley of materials has been harnessed for reinforcing dental composites. Diverse candidates, including metals, fibers, and an array of oxides such as aluminum, zirconium, and titanium, have been harnessed, yielding outcomes both auspicious and deleterious [9,10]. Recent endeavors have pivoted towards enhancing resin matrices through the infusion of nanofillers and particulates, ushering in the advent of nanocomposites endowed with enhanced properties [11]. The augmentation of filler content and, concomitantly, mechanical attributes has been orchestrated through the incorporation of micro-fillers [12] or the infusion of pre-polymerized resin fillers into the resin matrices of microfilled composites [13]. Copious empirical investigations have duly substantiated that the incorporation of various fillers, typically of micro and nano dimensions, begets a marked enhancement in the mechanical, physical, and biological attributes of dental resins [4-7]. Nonetheless, cautionary notes resound, as reports have surfaced elucidating inadvertent side effects, encompassing the genesis of voids and porosity, diminished biocompatibility, and the specter of gradual polymerization attenuation over time [10-14].

Biocompatibility is a fundamental aspect of dental materials, as any dental device or restoration that comes into contact with oral tissues must not elicit adverse reactions or compromise the health and well-being of patients. The intricate interplay between the chemical composition, physical properties, and biological response of 3D-printed dental resins necessitates a systematic evaluation to ensure patient safety and clinical efficacy. Hence, through the means of this systematic review, we aim to investigate the multifaceted realm of biocompatibility concerning 3D-printed dental resins and to comprehensively assess the existing body of literature to provide valuable insights into the biocompatibility profiles of these materials.

## Review

Methods

**Figure 1:**
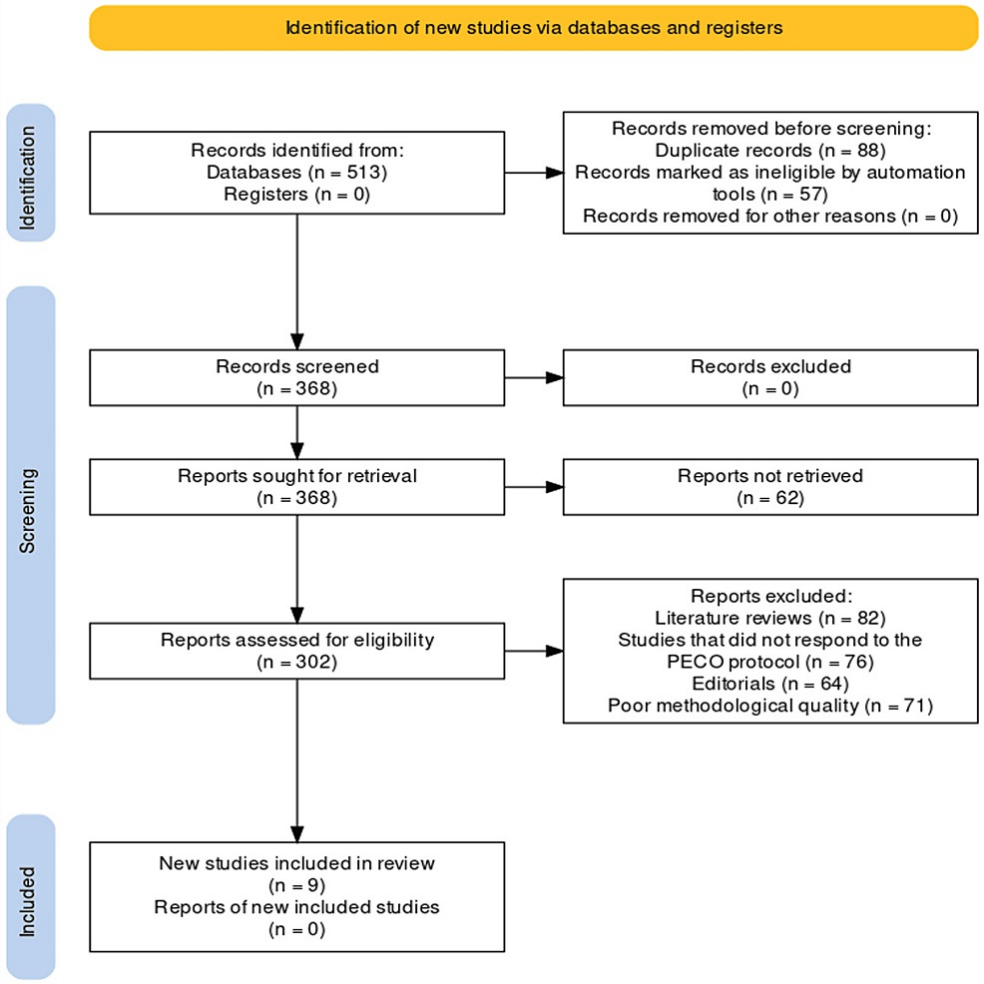
PRISMA protocol utilized for the review

This systematic review followed the Preferred Reporting Items for Systematic Reviews and Meta-Analyses (PRISMA) guidelines to ensure rigor, transparency, and replicability in the selection, screening, and inclusion of relevant studies [15]. The initial phase of study selection adhered to PRISMA guidelines, and an exhaustive search strategy was executed to identify potential studies elucidated further through Figure 1.

Moreover, the bias assessment protocol pertaining to this review was adapted from the modified Consolidated Standards of Reporting Trials (CONSORT) guidelines for dental materials and was systematically applied to ensure the reliability and validity of the review's findings [16].

The Population (including animal species), Exposure, Comparator, and Outcomes (PECO) framework for this review is presented in Table 1.

**Table 1:** PECO protocol devised for the review PECO: Population (including animal species), Exposure, Comparator, and Outcomes

Criteria	Description
Population (P)	Dental resins or materials intended for use in dental applications, specifically those that are 3D printed. The studies included in the review focused on these dental resins.
Exposure (E)	In-vitro or in-vivo assessment of the biocompatibility of 3D-printed dental resins. Assessments included mechanical properties, cytotoxicity, cell viability, surface characteristics, and other factors related to biocompatibility. Studies relevant to the review reported on the biocompatibility of dental resins when exposed to biological systems or simulated in-vitro conditions.
Comparator (C)	Comparisons of different dental resins within the same study or across different studies to evaluate relative biocompatibility. This could involve different resin formulations, manufacturing process variations, or varying parameters such as post-curing temperatures and material additives.
Outcome (O)	The primary outcome was the assessment of biocompatibility, which included cytotoxicity, cell viability, cell behavior, inflammatory responses, oxidative stress, and mechanical properties. Studies included in the review reported on these outcomes in either in-vitro laboratory settings or in-vivo biological contexts.

Search Protocol

PubMed/MEDLINE, Scopus, Web of Science, Embase, Cochrane Library, CINAHL, and Google Scholar were among the databases that were searched. The operators "AND," "OR," and "NOT" in Boolean logic were employed to efficiently combine search terms. MeSH keywords and their synonyms were utilised in order to guarantee a comprehensive selection of pertinent articles. Table 2 illustrates how the search strategy was modified to take into account the unique search syntax and capabilities of each database.

**Table 2:** Search strings utilized across the databases

Database	Search terms	Boolean operators	MeSH keywords	Search adaptations
PubMed/MEDLINE	(Dental resin* OR dental material* OR dental polymer* OR dental composite*) AND	AND	"Dental Restoration Materials"[Mesh] OR "Dental Materials"[Mesh] OR "Resins, Synthetic"[Mesh] OR...	Adapted to PubMed/MEDLINE syntax
Scopus	(Dental resin* OR dental material* OR dental polymer* OR dental composite*) AND	AND	"Dental Restoration Materials"[Mesh] OR "Dental Materials"[Mesh] OR "Resins, Synthetic"[Mesh] OR...	Adapted to Scopus syntax
Web of Science	(Dental resin* OR dental material* OR dental polymer* OR dental composite*) AND	AND	"Dental Restoration Materials"[Mesh] OR "Dental Materials"[Mesh] OR "Resins, Synthetic"[Mesh] OR...	Adapted to Web of Science syntax
Embase	(Dental resin* OR dental material* OR dental polymer* OR dental composite*) AND	AND	"Dental Restoration Materials"[Mesh] OR "Dental Materials"[Mesh] OR "Resins, Synthetic"[Mesh] OR...	Adapted to Embase syntax
Cochrane Library	(Dental resin* OR dental material* OR dental polymer* OR dental composite*) AND	AND	"Dental Restoration Materials"[Mesh] OR "Dental Materials"[Mesh] OR "Resins, Synthetic"[Mesh] OR...	Adapted to Cochrane Library syntax
CINAHL	(Dental resin* OR dental material* OR dental polymer* OR dental composite*) AND	AND	"Dental Restoration Materials"[Mesh] OR "Dental Materials"[Mesh] OR "Resins, Synthetic"[Mesh] OR...	Adapted to CINAHL syntax
Google Scholar	(Dental resin* OR dental material* OR dental polymer* OR dental composite*) AND	AND	"Dental Restoration Materials"[Mesh] OR "Dental Materials"[Mesh] OR "Resins, Synthetic"[Mesh] OR...	No specific syntax adaptation

Selection Criteria

Table 3 shows the inclusion and exclusion criteria that were devised with respect to the studies that were considered to be eligible for inclusion within this systematic review.

**Table 3:** Inclusion and exclusion criteria devised for the review

Criteria	Description
Inclusion criteria	
Study type	In-vitro and in-vivo studies investigating the biocompatibility of 3D printed dental resins, including experimental research in both laboratory settings (in-vitro) and living organisms (in-vivo).
Publication type	Peer-reviewed journal articles and conference proceedings, ensuring studies have undergone a formal review process.
Publication date	Studies published up to the date of the search, with no restrictions on the year of publication.
Exclusion criteria	
Irrelevant studies	Studies not directly addressing the biocompatibility of 3D printed dental resins, to maintain focus on the research question.
Literature reviews	Literature review articles, as the review was centered on original research studies.
Editorials	Editorial articles, opinion pieces, and commentaries, since they typically lack original research findings.
Poor methodological quality	Studies with significant methodological flaws or a lack of methodological transparency, to maintain high research quality standards.

Results

The structure for reporting this systematic review was provided by PRISMA, which we followed. Our review started with a thorough search of databases and registrations, which produced the identification of 513 records, in accordance with the PRISMA statement. We documented the number of records identified from each database and register in accordance with PRISMA's identification phase. We eliminated 88 duplicate records and disregarded 57 records that automated tools determined to be ineligible in accordance with the PRISMA screening procedure. Following these removals, 368 records moved on to the following stage in accordance with PRISMA rules for study selection. In accordance with the PRISMA eligibility requirements, we thoroughly assessed the 368 records. In order to verify that the research met our predetermined inclusion criteria, a comprehensive evaluation was conducted at this stage. Consequently, a total of 302 papers were extracted, out of which 62 were not recovered. We next applied PRISMA's exclusion criteria to eliminate 82 literature reviews, 76 studies that did not comply with our PECO procedure, 64 editorials, and 71 studies that had poor methodological quality. Ultimately, nine papers were found to be eligible and included in the systematic review, using the PRISMA inclusion methodology [17-25].

Through a retrospective analysis of differences across the PECO that was implemented in this study, we evaluated study heterogeneity. Dental resins used in 3D printing for dental purposes were the subject of the chosen studies. We assessed the mechanical characteristics, cytotoxicity, and other pertinent variables of these resins in order to determine their biocompatibility using both in-vitro and in-vivo techniques. Variations in the manufacturing procedures and resin formulations were used as benchmarks to assess the relative biocompatibility. Numerous biocompatibility outcomes, including cytotoxicity and cell viability, were the main outcomes that were examined. Peer-reviewed experimental investigations on 3D-printed dental resins were the main focus of the inclusion criteria, which were open-ended. To guarantee a high-quality, pertinent evidence base, studies that were not directly connected, literature reviews, editorials, or had subpar methodology were removed. Table 4 presents the overview of the objectives and parameters assessed in the included studies concerning biocompatibility assessments of different materials and methods in the context of 3D-printed resins [17-25]. These studies collectively contribute to the understanding of biocompatibility and related factors across a range of materials and applications.

**Table 4:** Characteristics and inferences drawn from the included papers CAD-CAM: computer-aided design and computer-aided manufacture; PMMA: polymethylmethacrylate; UA: urethane acrylate

Study	Objective focus	Evaluated metrics	Key findings	Summary insight
Alifui et al. [17]	Examine 3D printing materials' safety	- Composition analysis - Zebrafish embryo toxicity	- Ethanol treatment reduced material toxicity	Improved 3D printing safety through material selection and post-processing.
Alshamrani et al. [18]	Test dental resin enhancements	- Mechanical testing - Cellular assays - Surface analysis	- Added fillers increased strength and viability	Dental resin advancements suggest the potential for restorative applications.
Bayar et al. [19]	Optimize 3D-printed denture curing	- Mechanical and hardness tests - Cellular response	- Optimal strength and viability at higher curing temperatures	Curing conditions are crucial for dental resin performance.
Guerrero et al. [20]	Compare the biocompatibility of dental splints	- Cellular health assays - Microscopy	- Some resins reduced cell health, others comparable to control	Material choice is key in dental splint biocompatibility.
Hwangbo et al. [21]	Influence of wash protocols on dental resins	- Viability and toxicity tests - Mechanical assessments	- Longer wash times improved viability and decreased toxicity	Washing protocols impact resin biocompatibility and mechanical integrity.
Srinivasan et al. [22]	Contrast CAD-CAM and printed denture resins	- Biological and mechanical tests - Texture analysis	- Varying mechanical properties but similar biocompatibility	Manufacturing methods influence denture resin properties.
Tzeng et al. [23]	Assess UA-based resin parameters	- Viscosity and strength tests - Toxicity evaluation	- Customizable mechanical properties; non-toxic profiles	UA-resins offer tunable, safe options for 3D printing.
Ulmer et al. [24]	Examine 3D-printed resin properties	- Toxicity and mechanical testing - Statistical reliability analysis	- No significant toxicity post-7 days; variable mechanical performance	3D printed resins show potential, with PMMA leading mechanically.
Wuersching et al. [25]	Test printable resins for dental frameworks	- Viability and inflammatory response - Oxidative stress levels	- Varying toxicity levels; some increase in inflammatory markers	Printable resins vary in biocompatibility, influencing dental framework selection.

Alifui et al. discerned that ethanol post-treatment ameliorated the toxicity of the materials, thus enhancing the safety profile of 3D-printed objects [17]. This emphasis on post-processing as a determinant of material safety was not paralleled in the other studies, marking a distinct avenue for reducing toxicity in 3D-printed dental resins. Alshamrani et al. observed that the incorporation of fillers into dental resins augmented both the mechanical strength and the biological viability of these materials, indicating potential for restorative applications [18]. This contrasted with the work of Tzeng et al., who, although they highlighted the adaptability of mechanical properties in 3D-printed resins, did not link these properties to the integration of fillers, suggesting that the mechanical enhancements observed by Alshamrani et al. were specifically attributable to filler content [18,23].

Bayar et al. underscored the significance of curing conditions, demonstrating that elevated temperatures bolstered the strength and viability of the resins [19]. This insight into the role of curing conditions offered a novel perspective when juxtaposed with the findings of Ulmer et al., who reported on the potential of 3D-printed resins across a spectrum of mechanical performance outcomes without delving into the curing process [24]. The comparative analysis by Guerrero et al. shed light on the variable biocompatibility of dental resins, with some formulations impairing cellular health while others performed on par with controls [20]. This focus on the choice of resin material as pivotal for biocompatibility formed a dichotomy with the findings of Hwangbo et al., who emphasized the influence of wash protocols on the biocompatibility and toxicity of the resins, suggesting that pre-use processes are as critical as the material composition itself [21].

Hwangbo et al. also revealed that extended wash protocols could enhance the viability and reduce the toxicity of the resins, a complement to Alifui et al.'s post-processing insights, though distinct in that it concerned pre-use preparation instead of material modification [17,21]. In assessing the manufacturing methods, Srinivasan et al. noted that despite the differing mechanical properties between CAD-CAM and 3D-printed denture resins, biocompatibility remained consistent across the two [22]. This finding provided context for the observations of Wuersching et al., who documented variability in biocompatibility across resins, suggesting that manufacturing methods might interplay with biocompatibility outcomes [25].

The research by Tzeng et al. and Ulmer et al. both affirmed the non-toxic nature and potential of 3D-printed resins; however, their emphasis diverged, with Tzeng et al. accentuating the customizable nature of resin properties, and Ulmer et al. drawing attention to performance variability, thereby highlighting a distinction between the customization capabilities and performance consistency of these materials [23,24]. Wuersching et al. reported variable levels of toxicity and inflammatory response in different resins, underlining the heterogeneity in biocompatibility across 3D-printed dental materials [25]. This thread of variability is a recurring theme across the studies, with each research project addressing it from a unique perspective, be it material composition, manufacturing technique, or post-processing protocol.

**Figure 2:**
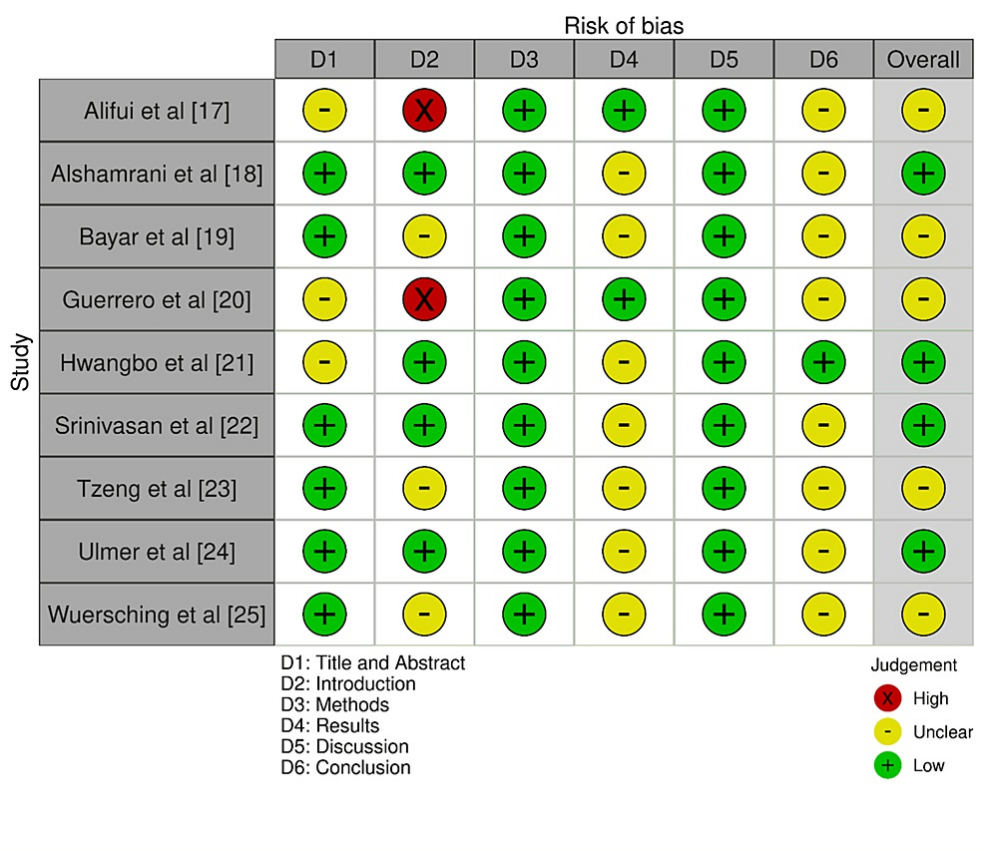
Bias evaluation in the selected papers

Figure 2 shows the bias assessment results of the included studies across different domains. The bias indicated an overall low to moderate level of bias across the six different domains as evident in Figure 2. This rating of bias helps to validate the reliability of the evidence presented in our review and supports the conclusions we have drawn from our findings.

Discussion

The implications of our findings are multifaceted, suggesting that future research and development should prioritize the enhancement of material safety, mechanical robustness, and biocompatibility. Enhanced safety protocols, including the post-processing of materials, emerge as a significant recommendation. Evidence indicates that treatments, such as ethanol washing, can markedly decrease the toxicity of 3D printing materials. Thus, standardized post-processing methods should be developed and integrated into the workflow to mitigate potential toxic effects. Mechanical property optimization is also a key area of focus. The integration of fillers and the fine-tuning of curing processes have been shown to improve strength and viability. Future research should aim to establish optimal composite material formulations and curing protocols that maximize the mechanical integrity of printed objects while maintaining or enhancing their functional properties.

The studies collectively underscore the necessity of tailoring 3D printing parameters-including material selection, manufacturing techniques, and post-processing-to specific application needs. This could lead to the development of application-specific guidelines that ensure the best possible outcome for the intended use, whether in dental applications or other areas of medicine. Furthermore, the variation in biocompatibility among different materials indicates that comprehensive biocompatibility assessments should become a standard part of the development process for new 3D printing materials. Such assessments would inform safer clinical practices and material choices, underpinning the personalization of treatments.

The influence of manufacturing methods on the properties of the final product also suggests that future research should explore the comparative effects of different 3D printing techniques and how they impact the clinical efficacy of the printed objects. The variability in biocompatibility and toxicity profiles among materials warrants a deeper investigation into the long-term effects of these materials on human health. This could result in the development of a new generation of resins and composites that are not only mechanically superior but also inherently non-toxic and less inflammatory, thus promoting better patient outcomes.

In the study by Alifui et al., the team investigated how 3D-printed materials interact with biological systems, examining both the materials' composition and their effects on living organisms through tests commonly used in environmental research [17]. Alshamrani et al. explored the physical robustness and biological safety of materials used in dental practices [18]. Their research was comprehensive, assessing the materials' strength and flexibility, their interaction with cells, and their detailed structural and elemental makeup, informing their suitability for dental use. Bayar et al. delved into the effect of different finishing processes on the physical and biological characteristics of 3D-printed dental products [19]. They meticulously measured strength, hardness, and cellular responses, revealing insights into how these post-production techniques can affect the dental products' performance. Guerrero et al. assessed various dental materials for their safety and potential effects on oral tissues, utilizing a range of tests to measure cell health and behavior, which helped to understand how these materials might interact with the body [20].

The effects of various cleaning agents and times on the qualities and safety of 3D-printed dental materials were examined by Hwangbo et al. [21]. Their evaluations covered a wide range of factors, such as surface imaging, material strength, and cell health, highlighting the significance of post-processing procedures on the mechanical and biological properties of the materials. The safety, mechanical characteristics, and surface roughness of resins that were 3D printed and CAD-CAM machined for complete dentures were assessed by Srinivasan et al. [22]. Their evaluations, which included texture analysis, strength tests, and biocompatibility testing, were helpful in comparing various production techniques for dental applications. Tzeng et al.'s study concentrated on the viscosity, strength, hardness, and cell safety of photopolymer resins used in 3D printing [23]. Their research demonstrated how these resins' mechanical characteristics might be changed without compromising their non-toxic characteristics.

Ulmer et al. investigated the safety and properties of 3D printer resin, with assessments that covered cell safety tests, strength measurements, and statistical reliability analysis [24]. Their study emphasized the mechanical qualities and safety of 3D printer resins, providing a foundation for their use in various applications. Wuersching et al. assessed the safety of printable resins used in dental prosthesis manufacturing, including parameters like cell health, inflammatory response, stress markers, and cell death [25]. Their study brought to light the different safety profiles and inflammatory potentials among various printable resins, shedding light on their appropriateness for dental prosthesis production.

Alifui et al. investigated the biocompatibility of 3D-printed photopolymers, highlighting the importance of material composition and its influence on biocompatibility [17]. The study's zebrafish assay revealed variations in toxicity among materials, and intriguingly, some materials exhibited reduced toxicity following ethanol treatment. This underscores the significance of material composition and post-processing methods as pivotal parameters for assessing the biological risks associated with 3D printing photopolymers. The study confirms the utility of zebrafish assays as a reliable tool for quantifying toxicity in additive manufacturing (AM) materials. Alshamrani et al. and Bayar et al. both examine the mechanical strengths of dental resins, with Alshamrani et al. finding improved flexural strength through additives and Bayar et al. identifying post-curing temperature as a key factor in mechanical enhancement [18,19]. Both studies also report on the biocompatibility of these resins, though Bayar et al. note a potential increase in cytotoxicity under certain post-curing conditions, a factor not addressed in the study by Alshamrani et al. [18,19].

Guerrero et al. and Hwangbo et al. focus on the biocompatibility of dental resins, with Guerrero et al. comparing various resins used in splints and finding similarities in biocompatibility profiles except for one outlier, while Hwangbo et al. emphasizes the importance of post-processing washing steps in improving biocompatibility [20,21]. Both studies underscore the critical nature of selecting biocompatible materials, though the parameters affecting this property differ between the two investigations. Srinivasan et al. look at both biocompatibility and mechanical properties like the previous studies but also include surface roughness in their analysis, offering a broader evaluation of material characteristics [22]. Their findings of similar biocompatibility across different manufacturing methods resonate with the general consensus of the other studies that many dental resins are biocompatible.

Tzeng et al. and Ulmer et al. both delve into the tunability of mechanical properties and biocompatibility, with Tzeng et al. demonstrating non-toxicity in urethane acrylate (UA)-based resins and the ability to adjust mechanical properties [23,24]. Ulmer et al. also observe variations in mechanical strength and cytotoxicity but specifically note the superiority of milled polymethylmethacrylate (PMMA) over 3D-printed resins in terms of mechanical strength. These studies highlight the importance of material selection for achieving desired properties and ensuring safety. Wuersching et al. [25], while also examining biocompatibility, look beyond cytotoxicity to include inflammatory responses, providing a more comprehensive view of the biological impact of dental resins. This study illustrates the complex nature of biocompatibility, encompassing a broader range of biological effects than what is typically measured.

In recent dental research endeavors, a paramount focus has converged upon the refinement of 3D-printed dental materials, specifically in the context of dental bridges and crowns, with a primary aim of facilitating their seamless integration into clinical practice. This undertaking centers on the enhancement of two pivotal facets: biocompatibility and durability [26-27]. The confluence of robust mechanical attributes and biological harmoniousness assumes particular prominence due to their potential ramifications on the enduring functionality of dental prostheses. Moreover, it is discerned that the material's quality and performance are inherently entwined with multifarious factors, spanning the degree of polymerization, the incorporation of fortifying agents, and the orchestration of printing parameters [28-30]. Hence, judicious scrutiny of these variables emerges as an imperative preliminary step in the judicious selection of dental materials. The mechanical competence of these materials, notably characterized by flexural strength, assumes a pivotal role in conferring resilience against the rigors of masticatory forces. This assumes paramount significance in the context of 3D-printed temporary restorations, which are often pressed into protracted service until the fabrication of the ultimate restoration [14,31].

One method that has gained significant traction in strengthening the flexural strength and several other properties of dental resin composites is the thoughtful use of fillers in the form of nanoparticles [14,32]. This approach, underpinned by empirical substantiation, extends its purview beyond augmenting flexural strength to encompass the augmentation of tensile strength, bolstering wear resistance [33-34], elevating elastic modulus, and mitigating polymerization shrinkage-a compendium of properties that collectively bolster the material's overall performance and viability [35]. In alignment with the investigations conducted by Alshamrani et al., the augmentation of flexural strength within 3D-printed resins has been distinctly correlated with the introduction of nanoparticles [18]. Evidently, this enhancement materializes in a pronounced manner with the inclusion of zirconia nanoparticles, manifesting conspicuous elevations when incorporated at concentrations of 10% and 20%, as well as with the introduction of glass fillers, whereby notable increments become discernible at concentrations of 5% and 10%. This corroboration substantiates the coherence of their findings with antecedent inquiries that have delved into the influence of diverse nanoparticulate additives on the mechanical attributes of 3D-printed resinous compositions [14,36]. It is noteworthy that these outcomes, although commendable, are situated within a comparable echelon to analogous investigations involving temporary resin-based materials. However, juxtaposed with antecedent investigations pertaining to flexural strength, it becomes discernible that the 3D-printed provisional material, fortified through the infusion of nanoparticles, tends to exhibit a somewhat diminished flexural robustness [30,37-38].

In the review conducted by Su et al., the emphasis was placed on the exceptional properties of AM zirconia in the field of dentistry [39]. The authors underscored its high mechanical performance, aesthetic appeal, and biological stability, which make zirconia a material of choice for personalized dental devices. The advantages of AM in producing complex structures that traditional methods struggle to achieve were also highlighted, with a particular focus on the accuracy and biocompatibility of 3D-printed zirconia. Additionally, the review by Su et al. provided insights into the current challenges facing AM zirconia and projected its future developments and improvements in oral medicine [39]. Contrastingly, the review by Gad et al. centered on the factors influencing the strength of 3D-printed resins, a different class of materials used in dentistry [40]. Their findings indicated that factors such as filler incorporation, printing orientation, post-polymerization conditions, and rinsing times had significant impacts on resin strength. The review concluded that the strength of 3D-printed resins could be enhanced through optimization of these factors, though it also called for further research to explore the combined effects of these variables.

The similarities between the two reviews, Su et al. and Gad et al. lie in their focus on the optimization of materials for dental applications through additive manufacturing technologies [39,40]. Both identify the critical role of material properties such as strength, biocompatibility, and the influence of manufacturing processes on these properties. However, the materials in question and their specific applications within dentistry are distinct. However, the dissimilarities are evident in the scope of the materials and the particular attributes each review examined. Su et al. dealt with zirconia, a ceramic with high mechanical stability, whereas Gad et al. reviewed resins, which are polymers that can be reinforced and manipulated in various ways to enhance their strength [39,40]. While Su et al. focused on the broader capabilities and potential of AM zirconia within oral medicine, Gad et al. presented a more granular analysis of the factors affecting a specific mechanical property of 3D-printed resins [39,40].

Della et al. presented a comprehensive systematic review of the state-of-the-art of restorative materials for 3D printing based on stereolithography [41]. Their research highlighted the impressive growth of stereolithography (SLA) in dentistry and its potentially disruptive impact on dental restoration methods. They noted that the majority of studies they reviewed utilized polymer-based restorative materials, with a focus on dimensional accuracy, strength, and surface morphology. Similar to our review, they found that there is considerable proof of concept work being done to showcase the clinical feasibility of SLA 3D printing for restorative materials, yet actual clinical applications in patients remain limited. This aligns with our findings, which might have also underscored the potential of AM technologies while recognizing the gap between laboratory research and clinical practice.

Pituru et al. focused on the biocompatibility aspects of PMMA-based materials used in interim prosthetic restorations, examining their interactions with the oral environment and the potential adverse effects [42]. They pointed out the complex interaction between PMMA materials and the oral cavity, which paralleled our review's concerns about material-environment interactions. Both reviews may have shared concerns about the biocompatibility and biochemical responses of the materials being studied, although our review might have also considered additional factors such as mechanical properties and long-term stability.

Limitations

It is important to take several limitations into account when interpreting the study's findings. First, the limited number of studies included in the systematic review may have restricted the breadth and depth of the analysis. While every effort was made to comprehensively search for relevant literature, the relatively small pool of eligible studies limits the generalizability of the conclusions. Additionally, the selected studies employed a variety of test methods, making direct comparisons challenging. Variations in study design, materials, and evaluation protocols further compounded the heterogeneity of the data. Another limitation relates to the diversity of dental resin formulations and 3D printing technologies. The inherent variability in the resin composition, as well as the use of different 3D printing platforms, introduces confounding factors that may have influenced the results. A more standardized approach to resin formulation and printing processes would facilitate more robust comparisons across studies. Additionally, the systematic review primarily focused on the biocompatibility of these resins, neglecting potential factors such as cost-effectiveness, ease of use, and clinical practicality, which are also critical for their successful integration into dental practice. Future research should consider a more holistic assessment that encompasses these aspects. Finally, while the included studies addressed several parameters related to biocompatibility, the broader context of clinical outcomes, patient-specific factors, and real-world dental applications was not within the scope of this review. Thus, the translation of these findings to clinical decision-making should be made cautiously, considering the complexities of dental treatments and individual patient characteristics.

## Conclusions

The collective findings suggest that while 3D-printed dental resins hold promise for various applications in restorative dentistry, there are notable variations in their biocompatibility profiles. The incorporation of additives such as zirconia and glass fillers has demonstrated significant improvements in flexural strength, offering enhanced mechanical properties that are valuable in clinical settings. Moreover, the influence of post-curing temperature and time on the mechanical and biological performance of these resins was evident, emphasizing the importance of optimized postprocessing for desired outcomes. However, challenges persist, particularly in the case of specific resin formulations like Freeprint® splint, which exhibit cytotoxicity in biocompatibility assays. This highlights the need for careful material selection and consideration of biocompatibility when using 3D-printed dental resins, with the Freeprint® splint serving as a cautionary example. Furthermore, this review underscores the importance of standardization in resin formulations and 3D printing processes to facilitate meaningful comparisons across studies. Additionally, the limited number of studies and their relatively short follow-up periods raise questions about the long-term durability and sustained biocompatibility of these materials, necessitating further investigation.
